# Hydroxy Acids, the Most Widely Used Anti-aging Agents

**Published:** 2012-01-04

**Authors:** Eskandar Moghimipour

**Affiliations:** 1 Department of Pharmaceutics, Nanotechnology Research Center and Faculty of Pharmacy, Ahvaz Jundishapur University of Medical Sciences, Ahvaz, IR Iran

**Keywords:** *Hydroxy Acids*, *Epidermis*

Aging process begins from birth and continues during the lifetime. Skin aging is the result of some reactions in dermis, epidermis, pigment cells, hair follicles, sebaceous glands, blood vessels and secondary organs in the skin layers. In this case, the thickness of epidermis, which is the outmost layer of the skin, decreases, while the stratum corneum layer that is the outmost layer of the epidermis thickens. The phenomenon in turn causes roughness and scaling and finally break down of the skin.Anti-aging medicine is a medical specialty founded on the application of advanced scientific and medical technologies for the early detection, prevention, treatment, and reversal of age-related dysfunction, disorders, and diseases (1, 2).

Hydroxy acids, also called fruit acids, are among non-organic acids which have been used in the treatment of skin disorders since about 40 years ago. They are some of the most widely used and studied anti-aging skincare compounds. Clinical trials have shown the effectiveness of these ingredients in reversing the effects of photoaging and improving wrinkles, skin elasticity, tone and hydration. Alpha hydroxy acid (AHA) and beta hydroxy acid (BHA) are two main classes of hydroxy acids. AHA is used in the treatment of several skin conditions such as acne, scar, pigmentation, skin dryness and wrinkles. They involve in metabolic pathways and essential cell cycles, including Krebs cycle, glycolysis and biosynthesis of serin. AHAs act on both the epidermal and the dermal levels. When applied to the skin, AHAs stimulate the exfoliation of epidermal cells in the stratum corneum by interfering with the ionic bonding between these cells. This results in the sloughing off dull and rough skin and promotes cellular renewal. Initially used for treatment of hyperkeratosis and other skin conditions affecting subcutaneous turnover, AHAs were found to promote softer, smoother skin, faded wrinkles, lightened age spots, and decreased blemishes. AHAs also improve the subcutaneous barrier function, increase epidermal proliferation and thickness, and restore hydration and pursiness through an increase in hyaluronic acid. The well-known benefits of AHA’s include exfoliation, moisturization, reduction of fine lines and wrinkles, collagen synthesis, firming and skin lightening. Glycolic acid is the smallest at AHA compounds, extracted from sugar. Formulations containing 10-15% GA are currently used topically, vaginally and rectally or as ophthalmic preparations for treatment at skin aging and hyper pigmentation due to sunlight. A negative side effect of AHAs may be a sensation of stinging or burning immediately after application, particularly on people with sensitive skin. New more lipophilic AHAs will be utilized more in the future, especially when targeting oily skins (3).

Beta Hydroxy Acids (BHAs), such as salicylic acid, are very similar to AHAs except for difference in their solubility. In the other hands, they are lipid-soluble in contrast to water solubility of AHAs. This structure allows them to penetrate into the skin through sebaceous follicles, making it appropriate for patients with oily skin and open comedones (3). In addition to prove anti-inflammatory effect of BHAs (e.g. salicylic acid), the skin irritancy effect of them have also been proved to be less than AHAs. Beta hydroxy acid found in skin-care products works best in a concentration of 1-2% (3, 4).
